# Inhibition of Neutrophil Extracellular Traps Formation by Cl-Amidine Alleviates Lipopolysaccharide-Induced Endometritis and Uterine Tissue Damage

**DOI:** 10.3390/ani12091151

**Published:** 2022-04-29

**Authors:** Wenxiang Shen, Ayodele Olaolu Oladejo, Xiaoyu Ma, Wei Jiang, Juanshan Zheng, Bereket Habte Imam, Shengyi Wang, Xiaohu Wu, Xuezhi Ding, Baohua Ma, Zuoting Yan

**Affiliations:** 1 Lanzhou Institute of Husbandry and Pharmaceutical Science, Chinese Academy of Agricultural Science, Lanzhou 730050, China; shane095@foxmail.com (W.S.); aygenesis2succeed@yahoo.com (A.O.O.); 18903865563@163.com (X.M.); gw18848970753@126.com (W.J.); sfazjs1228@sina.com (J.Z.); bekihi14@gmail.com (B.H.I.); wangshengyi@caas.cn (S.W.); wx.258.h@163.com (X.W.); dingxuezhi@caas.cn (X.D.); 2 College of Veterinary Medicine, Northwest A&F University, Yangling District, Xianyang 712100, China; 3 Department of Animal Health Technology, Oyo State College of Agriculture and Technology, Igboora 201103, Nigeria; 4 College of Veterinary Medicine, Inner Mongolia Agricultural University, Hohhot 010010, China; 5 Department of Veterinary Science, Hamelmalo Agricultural College, Keren P.O. Box 397, Eritrea

**Keywords:** endometritis, neutrophil extracellular traps, Cl-amidine, tissue damage, inflammation, lipopolysaccharide

## Abstract

**Simple Summary:**

The progression of several inflammatory diseases, including endometritis, has been proven to be enhanced by the process of neutrophil extracellular traps (NETs) formation in pathogenesis. Therefore, averting the formation of NETs could be an effective method to combat endometritis. Cl-amidine has been reported to treat certain inflammatory diseases, but its therapeutic role in combating endometritis remains an enigma as it may likely attenuate NETs formation. Hence, we investigate the potential of NETs formation in the pathogenesis of endometritis using rats and the probable therapeutic indices of Cl-amidine against endometritis. It was revealed that LPS induced endometritis in rats, and the formation of NETs was detected in the rats’ inflamed uterine tissues. The treatment with Cl-amidine showed the regression of LPS-induced endometritis and protection against uterine tissue damage. Cl-amidine effectively reduced the expression of specific proteins that could enhance the formation of NETs, thereby attenuating the inflammatory response to LPS-induced endometritis in rats. Our research showed that Cl-amidine possesses potentially therapeutic properties against endometritis.

**Abstract:**

Endometritis is a common disease that affects the production in dairy cows and leads to severe losses in the dairy industry. Neutrophil extracellular traps (NETs) formation promotes pathogenic invasions of the lumen of the tissue, leading to inflammatory diseases such as mastitis, pancreatitis, and septic infection. However, research that could show the relationship between NETs and endometritis is scarce. Cl-amidine has been shown to ameliorate the disease squealing and clinical manifestation in various disease models. In this study, we investigated the role of NETs in LPS-triggered endometritis in rats and evaluated the therapeutic efficiency of Cl-amidine. An LPS-induced endometritis model in rats was established and found that the formation of NETs can be detected in the rat’s uterine tissues in vivo. In addition, Cl-amidine treatment can inhibit NETs construction in LPS-induced endometritis in rats. Myeloperoxidase (MPO) activity assay indicated that Cl-amidine treatment remarkably alleviated the inflammatory cell infiltrations and attenuated the damage to the uterine tissue. The Western blot results indicated that Cl-amidine decreased the expression of citrullinated Histone H3 (Cit-H3) and high-mobility group box 1 protein (HMGB1) protein in LPS-induced rat endometritis. The ELISA test indicated that Cl-amidine treatment significantly inhibited the expression of the pro-inflammatory cytokines IL-1β, IL-6, and TNF-α. The NETs were determined by Quant-iT^TM^PicoGreen dsDNA kit^®^, which indicated that Cl-amidine significantly inhibited the NETs in rat serum. All results showed that Cl-amidine effectively reduced the expression of Cit-H3 and HMGB1 proteins by inhibiting the formation of NETs, thereby attenuating the inflammatory response to LPS-induced endometritis in rats. Hence, Cl-amidine could be a potential candidate for the treatment of endometritis.

## 1. Introduction

Endometritis is a common disease in post-partum cows caused by infection by pathogenic microorganisms, leading to a weakened immune response [1,2]. Endometritis affects 15% to 42% of dairy cows and is a major cause of infertility in dairy cows, seriously influencing the economic benefits of the dairy industry [2,3]. Although the underlying pathophysiology of endometritis is unknown, most endometritis treatments are focused on systemic antibiotics [4,5]. On the other hand, antibiotic overuse increases the likelihood of antibiotic residues in dairy products and can even result in major food safety issues [5,6]. Exploring novel ways to treat cow endometritis is thus a critical objective. Neutrophil recruitment has been implicated in playing an important role in the pathophysiology of endometritis [7,8,9]. It was hypothesized that inhibiting neutrophil infiltration could be beneficial in the resolution of endometritis by targeting specific adhesion molecules and reducing tissue damage in endometritis. Neutrophils play an important immune role in post-partum cows, and the impaired function of neutrophils has been associated with endometritis in post-partum cows [7]. Neutrophil extracellular traps (NETs) can protect neutrophils from infections induced by pathogens [10,11]. The pathophysiological components of endometritis have been described to include leukocyte recruitment and infiltration, purulent discharges, and tissue destruction [12,13].

The discovery of NETs, which provide a new microbial killing mechanism affecting the pathophysiology of different infectious disorders [11,14], is a significant recent development in understanding neutrophil function. Extracellular NETs are extrinsic structures made up of granule and nuclear neutrophil elements that trap and kill bacteria outside of the cell [10,13]. Galvanized neutrophils release nuclear materials that mix with granular contents and are discharged into the extracellular environment to form a net of DNA, nuclear proteins, and granular enzymes during quasi cell death [14,15,16]. Excessive NETs formation, as seen in acute lung injury [17,18], cystic fibrosis [19], asthma [20,21], psoriasis [22], thrombosis [23,24], and preeclampsia [25], has an unmistakably beneficial role during infection, as a deficiency in NETs production, especially in chronic granulomatous disease [26], or degrading the scaffold by bacterial DNase. The NETs formation has also been incriminated in the pathophysiology of the following infectious diseases: appendicitis [27], septic formation in the urinary tract [28,29], Crohn’s syndrome infection [30], generalized erythematosus [31,32,33,34], and circulatory vessel degeneration [35].

Most of the NETs components, such as neutrophil elastase (NE), histone, high-mobility group box 1 protein (HMGB1), DNA, and myeloperoxidase (MPO), are substances that cause inflammation in the body [36,37,38,39]. They are classic damage-associated molecular patterns (DAMP), which can quickly cause spontaneous immune attacks and cause severe inflammation, which is difficult to eliminate by the underlying immune system [38,39]. A common DAMP, HMGB1, MPO can induce cell necrosis and pyroptosis, resulting in inflammatory storms and tissue damage in animals [37,39].

High mobility group box 1 (HMGB1) is an inflammatory cytokine produced by immune cells and damaged cells. HMGB1 has been implicated in the pathophysiology of a variety of disorders, including infections, malignancies, and arthritis [40,41,42,43]. We hypothesized that HMGB1 could become a new therapeutic target for a variety of disorders. Evidence suggests that HMGB1 is implicated in the development of endometritis [44,45] and that endometritis causes HMGB1 to be increased in uterine tissues, serum, and uterine secretions; when endometrial inflammation occurs, HMGB1 is inhibited, and HMGB1 is reversed [44]. HMGB1 could be used as a sensitive biomarker for severe inflammatory responses in treatment. MPO, a heme protein secreted by leukocytes, is one of the most studied and plays a key role in cellular inflammation and oxidative stress [46,47,48]. Through the generation of microbicidal reactive oxidants, MPO has become increasingly recognized as an essential component of the innate immune system [47]. At the same time, it has an effect on the arterial endothelium through a variety of mechanisms, including a change in NETs cellular cholesterol flux and a reduction in NO-induced circulatory stress relief [46,48].

Cl-amidine is an inflammatory inhibitor that ameliorates the disease course and clinical manifestation in various inflammatory disease models [49,50,51,52,53]. Cl-amidine has been shown to reduce the production of pro-inflammatory cytokines in a mouse model of fatal sepsis and mastitis [50,51]. Cl-amidine has been shown to reduce the production of NETs and tissue damage in severe acute pancreatitis [54,55,56]. However, the role of Cl-amidine in LPS-induced endometritis is yet unknown. During endometritis, excessive cytokine synthesis is harmful to cells, tissues, and organs, and reducing the cytokine storm protects against inflammatory damage. Cl-amidine may inhibit the synthesis of pro-inflammatory cytokines (IL-, IL-6, and TNF-) in LPS-induced endometritis, implying that it has therapeutic potential in the prevention of NETs and the treatment of chronic endometritis. The goal of this study was to verify how Cl-amidine affected LPS-induced rat endometritis and to discover the potential anti-inflammatory molecular mechanism.

## 2. Materials and Methods

### 2.1. Experimental Animals and Management

A total of 20 female 9–10 week-old SD rats (weighing 180–200 g) were purchased from the Experimental Animal Center, Lanzhou Veterinary Research Institute of the Chinese Academy of Agricultural Sciences (CAAS) (Lanzhou, China) and participated in all experiments. All rats were placed in a room with constant temperature (25 ± 1 °C) and humidity (50–68%) and provided with free access to food and water. All protocols for the use of animals were conducted following the NIH guidelines for animal use and care and were approved by the Animal Care and Use Committee of the Lanzhou Institute of Husbandry and Pharmaceutical Science of CAAS (approval number: NKMYD201907018).

### 2.2. Endometritis Model Induced by LPS and Experimental Design

The rats were divided into four groups of five rats per group. Rats were anesthetized with 10% chloral hydrate via intraperitoneal (IP) injection prior to specific experimental treatment. Before intrauterine injection, we cut off the sharp part of the tip of the 1 mL pipette gun tip (about 1 cm) with scissors as a rat vaginal dilator and inserted it into the rat vulva. Then, a blunt needle with a length of 50 mm was slowly inserted into the rat uterus. Later, a syringe containing LPS (1 mg/mL, dissolved in saline) was mounted, and LPS (1 mg/kg, Sigma, Burlington, MA, USA) was slowly injected into the uterus. The rat endometritis model was observed after 24 h. The rats were sacrificed through cervical dislocation, and uterine tissues were collected.

To understand the effect of inhibition of NETs formation on endometritis, rats were divided into 4 groups with 5 rats per group: (1) control, (2) LPS, (3) LPS + Cl-amidine, and (4) Cl-amidine. Cl-amidine (25 mg/mL, MedChem Express, 50 mg/kg, Monmouth, NJ, USA) or saline was administered 1 h before induction of endometritis and 23 h after the LPS challenge. All rats were euthanized in a cervical dislocation 24 h after the LPS challenge, the uterus was extracted intact, and the pathological alterations were photographed. To assess tissue damage and inflammation, tissue samples (approximately 0.5 cm) from the middle side of 2different uterine horns were fixed in 4% paraformaldehyde for 24 h, then dehydrated and embedded in paraffin wax. The paraffin-embedded tissue was cut into 8um-thick sections, stained with H&E, and observed with a light microscope. The severity of endometritis was assessed by examining the endometrial epithelium for disorganization and for leukocyte infiltration.

### 2.3. Enzyme-Linked Immunosorbent Assay

The uterine tissue was stored at −80 °C and was weighed at 0.1 g into a 2-mL tube, mixed with 500 μL of RIPA lysis buffer (Solarbio, Peking, China) containing 1% protease inhibitor cocktail I (Medchem express, Monmouth, NJ, USA), and ground with a tissue homogenizer. Grinding was performed until no intact tissue fragments were visible, and all steps of the grinding process were performed on ice. The supernatant was collected, centrifuged, and stored in a −20 °C refrigerator (15,000× *g*, 4 °C, and 10 min). The levels of interleukin-6 (IL-6), tumor necrosis factor-α (TNF-α), and interleukin-1β (IL-1β) were measured in the supernatant of uterine tissue homogenates using ELISA kits (Jinma, Shanghai, China) according to the manufacturer’s instructions. Absorbance was obtained at 450 nm using a Multiskan™ GO full-wavelength microplate reader (Thermo Fisher Scientific, Waltham, MA, USA).

### 2.4. Plasma NETs Quantification

To assess the amounts of circulating cell-free DNA (cf-DNA), blood was taken from the inferior vena cava and diluted (1:10) in acid citrate dextrose. The samples were centrifuged at 15,000× *g* for 15 min at 4 °C, and cf-DNA was measured using a Quant-iTPicoGreen dsDNA kit (Invitrogen, Carlsbad, CA, USA), as directed by the manufacturer. The fluorescence intensity of NETs was detected by a Multimode plate reader (Enspire, PerkinElmer, Chicago, IL, USA) at excitation and emission wavelengths of 480 nm and 530 nm, respectively.

### 2.5. Immunohistochemistry

Immunohistochemistry was performed according to the following procedure. Paraffin sections (8 μm) of uterine tissue were dewaxed and then rehydrated. Antigen extraction was performed in an autoclave at 110 °C for 15 min with an antigen repair solution. Rabbit polyclonal antibody anti-MPO (1:200, Proteintech, Chicago, IL, USA) was incubated for 1.5 h, followed by the addition of appropriate biotinylated secondary antibody and incubation for 30 min at room temperature, followed by color development with DAB kit (ZSGB-Bio, Beijing, China). The DAB chromogenic solution was washed away, then stained with hematoxylin, dehydrated, and transparent with xylene, and the sections were sealed with neutral resin.

### 2.6. Immunofluorescent Assay

Confocal microscopy was used to evaluate NETs formation in paraffin-embedded uterine tissue samples. From the paraffin-embedded uterine tissue, an 8 μm thick piece was cut and stained. Briefly, before antigen retrieval, paraffin sections were dewaxed and hydrated, heated in a pressure cooker with Tris-EDTA HIER(Heat-Induced Epitope Retrieval)solution for 2 min at 110 °C, pH = 9. Sections were blocked with Odyssey^®^ blocking buffer and stained at room temperature with a 1:200 dilution of rabbit anti-neutrophil elastase (NE) polyclonal antibody (AB68672, Abcam, Cambridge, UK) and 1:200 dilution of mouse anti-histone 1 monoclonal antibody (MAB3864, Millipore, Burlington, MA, USA) for 1.5 h in a humid chamber. After washing, Alexa Fluor 594 goat anti-mouse IgG (H+L) (1:100, SA00013-3, Proteintech, Chicago, IL, USA) and Alexa Fluor 488 goat anti-rabbit IgG (H+L) (1:50, SA00013-3, Proteintech, Chicago, IL, USA) were used as the secondary antibody, incubated for 1 h at room temperature. The final staining was done with Hoechst 33342 (C0030, Solarbio, Beijing, China) for 5 min, protected from light, and confocal images were taken with a Zeiss LSM 800 confocal microscope.

### 2.7. Western Blotting

Total protein was extracted from rat uterine tissue using Mammalian Protein Extraction Reagent (Solarbio, Beijing, China). BCA protein assay kit (Takara, Dalian, China) was used to determine the protein concentration. Based on the results of the BCA assay, the protein content of each group was adjusted to the same concentration using sterile water. Total proteins were denatured in a metal bath at 100 °C with a 5× loading buffer (Solarbio, Beijing, China) for 15 min. Total proteins (30 μg) were separated by 10% sodium dodecyl sulfate-polyacrylamide (SDS-PAGE) gel (Solarbio, Beijing, China) and transferred to the PVDF membrane (Solarbio, Beijing, China). The membranes were blocked with Quickblock^TM^Western Blocker (Beyotime Biotechnology, Shanghai, China) for 15 min at room temperature and then incubated with primary antibody at 4 °C overnight. Protein bands were observed with the ECL chemiluminescence kit (Thermo Fisher, Waltham, MA, USA) and detected with the G: BOX Chemi XRQ Gel Doc System (Syngene International Limited, UK). The primary antibodies used were as follows. (1:1500, 22225-1-AP, Proteintech, Chicago, IL, USA), anti-HMGB1 antibody (1:1000, 10829-1-AP, Proteintech, Chicago, IL, USA), anti-citH3 antibody (1:1000, ab281584, Abcam, Cambridge, UK), anti-β-actin antibody (1:2000, ab8226, Abcam, Cambridge, UK).

### 2.8. Statistical Analysis

Data were expressed as mean ± SD and analyzed using Graphpad prism 7 software (GraphPad Prism, San Diego, CA, USA). One-way analysis of variance (ANOVA) and unpaired *t*-test were used for comparison between groups. Western blots and immunohistochemistry results were analyzed using Image J software (National Institutes of Health, Bethesda, MD, USA). *p*-values less than 0.05 were considered significant, and *n* represents the number of animals.

## 3. Results

### 3.1. NETs Regulates Tissue Damage in Endometritis with Inhibition upon Cl-Amidine Treatment

**Figure 1:**
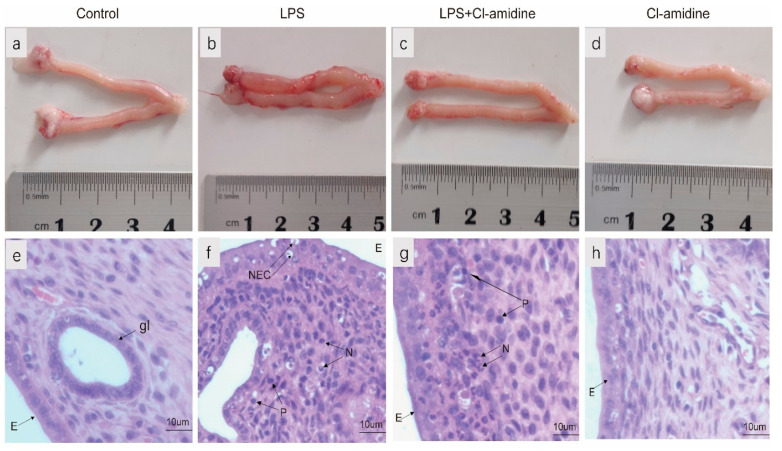
Anatomical changes and characterized pathological change in uterus inflammation. Anatomical changes in (**a**) control, (**b**) LPS treated group, (**c**) LPS + Cl-amidine treated group, and (**d**) Cl-amidine treated group. Characterization of pathological sections of (**e**) control, (**f**) LPS, (**g**) LPS + Cl-amidine, and (**h**) Cl-amidine. Cells labeled as N, neutrophil; NEC, necrosis epithelial cell; P, plasmacyte. The letter E indicates Epithelial Cells, whereas the letter gl indicates gland. Magnification 400×.

The normal rat uterus had a pale pink appearance (Figure 1a), and after H&E staining, the endometrial epithelial cells were microscopically seen to be intact, without necrotic epithelial cells, and without leukocytes in the uterine tissue (Figure 1e). LPS challenge caused severe disruption of uterine tissue structure, as evidenced by uterine edema and hyperemia (Figure 1b), endometrial epithelial cell necrosis, and leukocyte infiltration (Figure 1f). Inhibition of NETs formation significantly reduced LPS-induced uterine edema and hyperemia (Figure 1c), endometrial epithelial cell necrosis, and leukocyte infiltration in the uterus (Figure 1g). In addition, Cl-amidine administration alone did not affect the histopathology and morphology of the uterus (Figure 1d,h). This result revealed that Cl-amidine has protective activities against uterine inflammation.

### 3.2. Effect of Cl-Amidine on the Production of Pro-Inflammatory Cytokines in Uterus Tissues during LPS-Induced Endometritis in Rats

**Figure 2:**
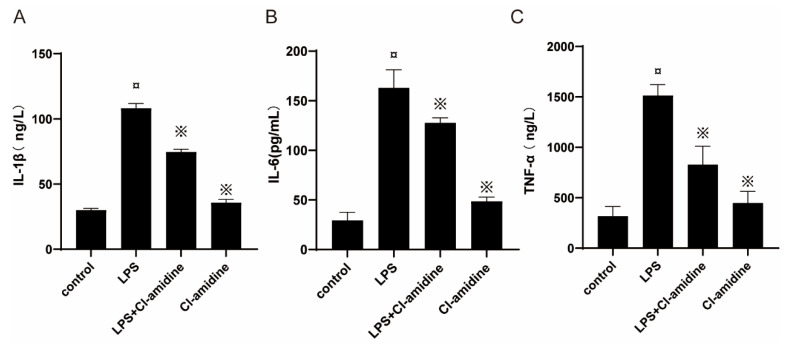
Effects of Cl-amidine on the mRNA levels of pro-inflammatory cytokines in the LPS-induced rat endometritis. Endometritis induced by infusion of LPS (1 mg/kg body weight) into the uterine cavity, later treated with Cl-amidine or vehicle (saline) as required. Samples were collected 24 h after induction of endometritis and Cl-amidine treatment. The mRNA levels of (**A**) IL-1β, (**B**) IL-6, and (**C**) TNF-α were measured by RT-PCR. Data were represented by mean ± SD (*n* = 5 animals per group). ¤ *p* < 0.05 vs. control rats and ※ *p* < 0.05 vs. LPS without Cl-amidine.

Increased levels of pro-inflammatory compounds in uterine tissues are characteristic of uterine inflammation in rats with endometritis. The levels of IL-1β, IL-6, and TNF- were found to be low but detectable in the uterine tissue of control rats. In contrast, the LPS challenge increased IL-1β levels in uterine tissue by 3.6-fold (i.e., from 30 to 108 ng/L). Administration of Cl-amidine reduced IL-1β levels in uterine tissue by 31% (Figure 2A). In addition, IL-6 levels were found to be greatly increased in uterine tissues of rats with endometritis compared to control rats, and interestingly, in the group with endometritis, treatment with Cl-amidine resulted in a 22% decrease in IL-6 levels in uterine tissue (Figure 2B). Injection of Cl-amidine reduced LPS-induced TNF-α levels in uterine tissues by 45%, and Cl-amidine injection alone did not affect IL-Iβ, IL-6, and TNF-α levels in uterine tissues (Figure 2C).

### 3.3. NETs Formation in LPS-Induced Endometritis and Effect of Cl-Amidine

**Figure 3:**
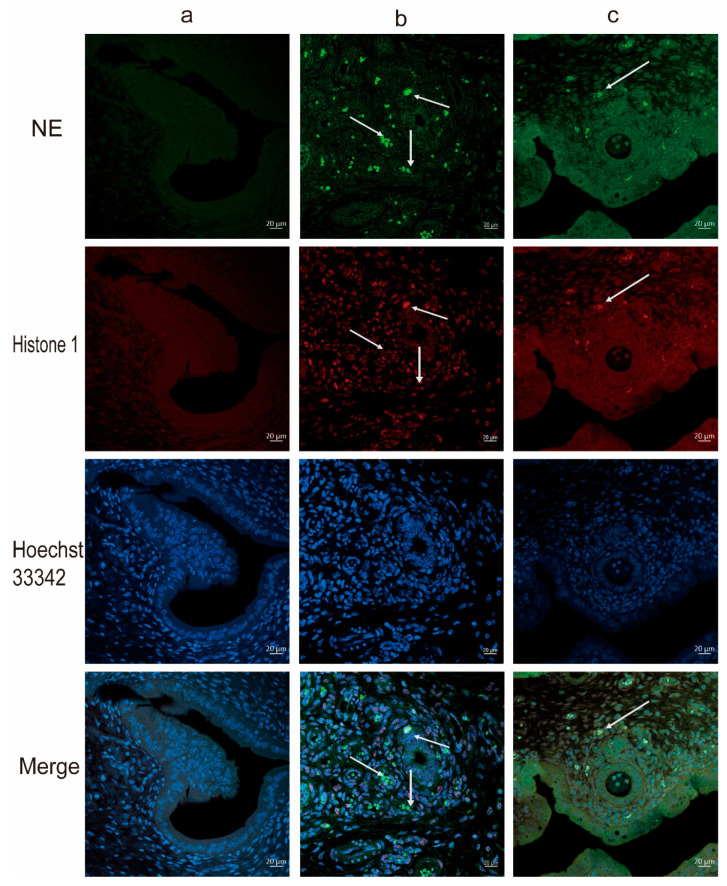
Neutrophil extracellular traps (NETs) in LPS-induced endometritis uterus but absent in healthy tissue, and decreased by treating with Cl-amidine. (**a**): control group. (**b**): the LPS group. (**c**): the LPS + Cl-amidine group. NE stands for neutrophil elastase. The red scale length is 20 um. The arrows are showing the NETs formation.

**Figure 4:**
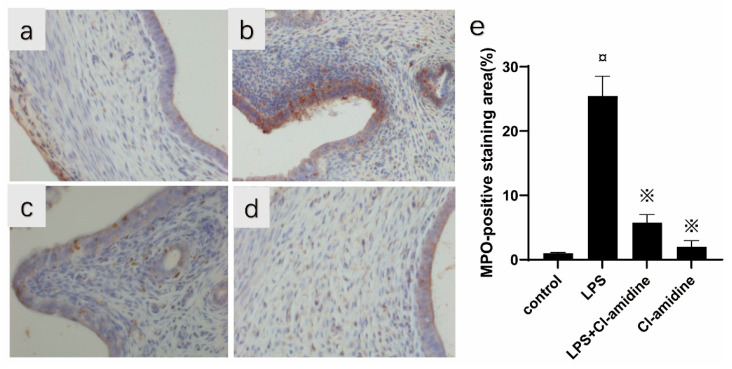
Immunohistochemical staining results of uterus tissue. Representative images of Myeloperoxidase expressing neutrophil infiltration by immunohistochemical staining in (**a**) control group, (**b**) LPS group, (**c**) LPS + Cl-amidine group, and (**d**) Cl-amidine group. (**e**) Percentage area of positive MPO staining for the above four groups. Data were represented by mean ± SD (*n* = 5 animals per group). ¤ *p* < 0.05 vs. control rats and ※ *p* < 0.05 vs. LPS without Cl-amidine. The dark yellow color indicates positive MPO, indicating the presence of neutrophils, and the light blue color is the nucleus.

**Figure 5:**
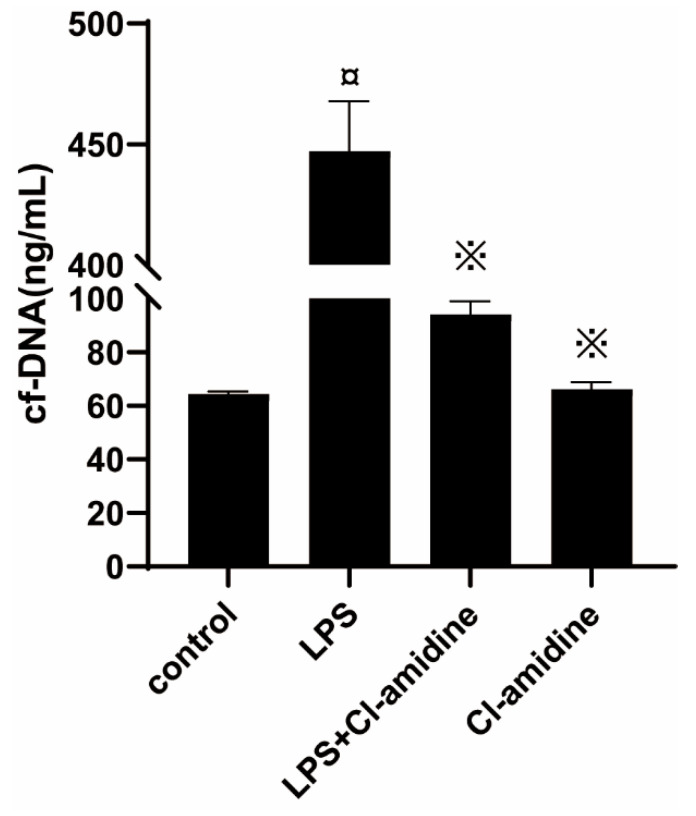
Quantification of extracellular DNA in the plasma of rats with LPS-induced endometritis with Cl-amidine treatment. Data were represented by mean ± SD (*n* = 5 animals per group). ¤ *p* < 0.05 vs. control rats and ※ *p* < 0.05 vs. LPS without Cl-amidine.

NETs are composed of extracellular DNA, granule proteins, and chromatin proteins from neutrophils. Thus, we found by immunofluorescence staining that the LPS challenge induced co-localization of neutrophil-derived granule protein (NE) as well as chromatin protein (histone 1) with extracellular DNA, indicating that NETs form in the inflamed uterus (Figure 3). Furthermore, with myeloperoxidase expressing neutrophil infiltration by Immunohistochemical staining, we found that, observed in normal uteri, the levels of neutrophils infiltrations were very minimal (Figure 4a) compared to massive neutrophils infiltrations in uteri treated with LPS (Figure 4b) or in uteri of LPS-exposed rats pretreated with Cl-amidine (Figure 4c) and few cellular infiltrations in the Cl-amidine alone treated group (Figure 4d). Moreover, the levels of cf-DNA were significantly higher in the LPS-induced endometritis model compared to those of rats treated with Cl-amidine in the endometritis model and normal controls (Figure 5). Therefore, the administration of Cl-amidine reduced the infiltration of neutrophils in the uterine tissue.

### 3.4. Effect of Cl-Amidine on NETs- Derived Proteins in LPS-Induced Rat Uterine Tissues

**Figure 6:**
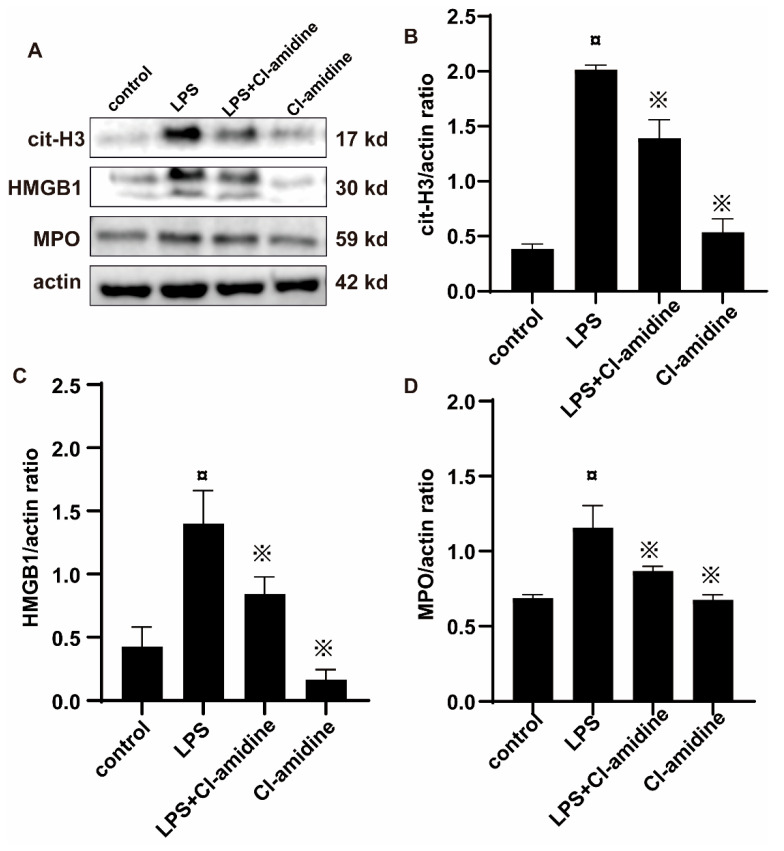
Effect of Cl-amidine on NETs-derived proteins in LPS-induced rat uterine tissues. The rats were grouped into four groups of five animals each. The control group was given normal saline, and groups 2, 3, and 4 were treated with LPS, Cl-amidine and LPS, and Cl-amidine, respectively. After the treatment, protein was extracted from the uterine tissues, and the protein expression was determined using the Western blot technique. (**A**) shows the expression blot of NETs-derived proteins were expressed, including cit-H3, HMGB1, and MPO with different molecular weight. (**B**) The graphs show the expression of cit-H3 in the different treatment groups. (**C**) The graphs show the expression of HMGB1 in the different treatment groups. (**D**)The graphs show the expression of MPO in the different treatment groups. Gray values of figure were analyzed by Image J. The values given are mean ± SD (*n* = 5 animals per group). ¤ *p* < 0.05 vs. control rats and ※ *p* < 0.05 vs. LPS without Cl-amidine.

Citrullinated histone H3 (Cit-H3) is an important marker for the formation of NETs. To assess the impact of Cl-amidine on the formation of NETs and whether they could be detected in uterine tissues, we examined the expression of Cit-H3 through Western blotting. As shown in Figure 6A,B, the expression of Cit-H3 in uterine tissues was significantly increased in the LPS stimulated group, while it decreased in the Cl-amidine-treated group. HMGB1 plays an important role in the pathogenesis of various diseases and is highly expressed in the uterus; its expression in the uterine tissues was significantly increased in the LPS stimulated group, while it decreased upon treatment with Cl-amidine (Figure 6A,B). MPO is most abundantly expressed in neutrophils stored in neutrophil azurophilic granules, often used as an indicator of neutrophil infiltration. We found that challenge with LPS increased uterine MPO activity by 41%, while Cl-amidine reduced LPS-provoked endometritis levels of MPO by 25% (Figure 6A,D).

## 4. Discussion

Endometritis affects cattle post-partum and decreases dairy industry profitability [1,2,57]. Dairy cattle have been the subject of intense genetic selection for milk production over the last 60 years resulting in susceptibility to uterine disease and decreased fertility [3,4,5,58]. Usually, cows can clear bacteria from the uterus and resolve inflammation before the third week post-partum. Inappropriate immune response activation early post-partum perturbs tissue remodeling, delays bacterial clearance, and prolongs endometrial inflammation [5,6]. Endometritis is a common cause of infertility in dairy cows, leading to incalculable economic losses [2,57]. The high number of neutrophils characterizes inflammation of the endometrium that significantly reduces reproductive performance without any signs of clinical endometritis [6,7]. Several bacteria have been reported to be involved in the pathogenesis of endometritis [2,4]. *Escherichia coli* is the most common pathogen for endometritis [4,6]. Lipopolysaccharide (LPS) is the endotoxic component of *Escherichia coli* and can stimulate inflammatory response [59,60], leading to the production of pro-inflammatory cytokines such as tumor necrosis factor (TNF-α) interleukins (IL-6, IL-1β). LPS is an important component of the outer membrane of Gram-negative bacteria, and it can promote the formation of NETs in mice [59,61,62]. LPS enters the body and accumulates the neutrophils to release inflammatory cytokines. LPS-induced endometritis animal models have been widely used to evaluate endometritis and other inflammatory diseases [63]. In this study, the inflammatory model was established, which has been frequently used to explore the anti-inflammatory mechanisms of drugs. Our results showed that Cl-amidine administration significantly attenuated the pathological changes in the uterus. Cl-amidine treatment decreased inflammatory conditions, including inflammatory cell infiltration, uterine cavity effusion, hyperemia, uterine epithelial cell detachment, and epithelial cell necrosis; there was an observed massive leukocyte infiltration in the non-treated inflammatory group, implying that NETs are involved in endometritis tissue damage. Thus, NETs formation is important in the pathophysiology of endometritis, as corroborates the earlier report [7,8,59]. Hence, focusing on NETs attenuation could be a good way to reduce tissue damage in the inflamed uterus and provide good therapeutic insight. In the condition of excessive or insufficient clearance, it is widely recognized that NETs can promote the development of inflammation. Neutrophil plays an important role in the process of endometritis, although their potential mechanism is unclear. This study shows that neutrophil-derived NETs form a central component of the pathophysiology of endometritis. In addition, we found that rats with endometritis showed increased levels of NETs components in their uterine tissue. Therefore, therapeutic strategies that target NETs formation may provide clinical benefits for animals with endometritis.

NETs are ancient defense weapons that have been conserved throughout evolution to capture and destroy microbes in the extracellular environment, allowing lower animals without an effective adaptive immune system to avoid infection [10,11,12,35,36,37,38,64,65]. However, NETs may be a double-edged sword in higher vertebrates, and a growing number of findings suggest that NETs cause tissue damage and exacerbate inflammatory responses in many inflammatory diseases, such as COVID-19 pneumonia [66] and inflammatory bowel disease [67].

After activating the innate immune system by LPS, strong pro-inflammatory signals are produced; these signals are important for maintaining a stable balance in the immune system and protecting the host from harmful effects [45]. Excessive pro-inflammatory cytokine release increases the level of the immune response, leading to an inflammatory cascade and tissue damage [63]. Therefore, inhibition of the release of inflammatory cytokines may serve as a target for anti-inflammatory drug development. We examined the uterine levels of IL-1β, IL6, and TNF-α across the experiment group and found that the LPS challenge significantly increased the tissue levels of IL-1β, IL-6, and TNF-α. Notably, the LPS-induced tissue levels of IL-1β, IL6, and TNF-α significantly decreased with Cl-amidine treatment, indicating that inhibition of NETs formation in the process of endometritis could be advantageous in protecting uterine tissue from damage and ultimately altered endometritis.

Furthermore, our study showed that induction of endometritis leads to DNA deposition in the inflamed uterus with an increased cf-DNA in plasma, which co-localizes with the neutrophil-derived granule proteins neutrophil elastase (NE) and histone. LPS significantly increased the level of NETs in rat serum. After Cl-amidine treatment, LPS-induced NETs in rat serum were significantly inhibited.

Cl-amidine is a broad peptidylarginine deiminase 4(PAD4) inhibitor, which can inhibit NETs formation by blocking histone citrullination in the nucleus of neutrophils [48,49,50,59]. Many studies have shown that pharmacological inhibition through Cl-amidine is an effective way to stop NETs formation in vitro and in vivo [48,49,50,51]. This view is supported by data from several studies in which NETs-derived DNA, granule proteins, and the chromatin proteins cit-H3 and HMGB1 were significantly elevated in inflammatory diseases. This theory is supported by evidence that inflammatory diseases lead to higher cf-DNA levels in plasma and of proteins such as NE and HMGB1 in tissues, both of which are components of NETs [40,41,42,43,44,45,68]. From this perspective, it was noteworthy that a recent study found that NETs-derived HMGB1 can directly cause epithelial damage [69]. In light of this finding, we found that NETs degradation by Cl-amidine treatment significantly reduced uterine cit-H3 and HMGB1 levels. It can serve as a pointer to preventing the NETs formation in endometritis is therapeutically important. Although the role of NETs in host defense against microbial infection is debatable [43], our study first reports that excessive NETs production appears to be detrimental to endometritis and invariably added endometritis to the list of infectious diseases such as sepsis and bacterial pneumonia, which NETs production implicated. Neutrophil infiltration is a typical characteristic of endometritis, with increased levels of myeloperoxidase (MPO) commonly used to suggest neutrophil infiltration [42,46,48]. In addition, neutrophil count is the classic diagnostic method for determining endometritis in cows. This study found that the LPS challenge increased MPO levels and neutrophil numbers in the inflammatory uterus. Cl-amidine treatment significantly reduced MPO levels and the number of neutrophils in uterine tissues, implying that NETs are an important regulator of neutrophil recruitment in the uterus. Given the importance of neutrophils in the pathophysiology of endometritis, the tissue-protective effect of preventing NET formation may be due, at least in part, to a reduction in neutrophil infiltration in the inflamed uterus. In the condition of excessive or insufficient clearance, it is widely recognized that NETs can promote the development of inflammation. Therefore, inhibition of NETs formation could be one of the therapeutic strategies for treating endometritis.

In conclusion, this study demonstrates that NETs are produced and play an essential role in the development of endometritis. In the inflammatory uterus, inhibiting NETs reduced MPO production and neutrophil recruitment. Furthermore, we show that NETs regulate the release of cytokines in the uterus. These findings point to a new role for NETs in endometritis and imply that targeting NETs could be a beneficial method to reduce local endometritis inflammation. This study demonstrates that Cl-amidine can alleviate LPS-induced rat endometritis by inhibiting NETs formation, blocking histone citrullination in the nucleus of neutrophils with reduced production of MPO. Our observations suggest that NETs inhibition using Cl-amidine could be further explored as a possible therapeutic maneuver against the damaging inflammatory response seen in endometritis, especially in high-producing dairy cows. Therefore, the need for future use of Cl-amidine in dairy cow endometritis could further affirm its neutrophil extracellular trap attenuation potential and be helpful in the regression of endometritis.

## Data Availability

Full data available from the first author.
